# Multisystemic Impairments in 93 Chinese Patients With Myotonic Dystrophy Type 1

**DOI:** 10.3389/fneur.2020.00277

**Published:** 2020-04-21

**Authors:** Mao Li, Zhanjun Wang, Fang Cui, Fei Yang, Hongfen Wang, Xusheng Huang

**Affiliations:** ^1^Department of Neurology of the First Medical Center, Chinese PLA General Hospital, Beijing, China; ^2^Department of Neurology, Xuanwu Hospital, Capital Medical University, Beijing, China

**Keywords:** myotonic dystrophy type 1, MIRS, multisystemic impairment, cataract, hypogonadism, cardiac abnormalities

## Abstract

**Background:** Myotonic dystrophy type 1 (DM1) is an autosomal dominant neuromuscular disease characterized by muscle weakness and multisystemic impairments, which significantly impact the quality of life. There is currently an increasing consensus on the necessity of a multidisciplinary assessment in patients with DM1, to improve the management of the disease.

**Methods:** To analyze the prevalence and pairwise relationships between various organs involved, we performed a retrospective study by reviewing demographic and clinical information of DM1 patients including age, disease duration, clinical history, muscular impairment rating scale score (MIRS), results of blood biochemistry, electrocardiogram, echocardiography, and ophthalmologic examination.

**Results:** Ninety three DM1 patients (60 males and 33 females), aged 34.7 ± 12.6 (mean ± standard deviation) years were recruited. Of which, two congenital cases were of maternal and paternal inheritance, respectively. In the other 91 patients, cataract was found in 44.1% of patients, followed by hypogonadism (40.8%), frontal balding (40.7%), and cardiac abnormalities (34.5%). Thyroid dysfunction and insulin insensitivity were relatively uncommon. Age (*p* < 0.001) is independently correlated with cataract, and MIRS correlates positively with cardiac abnormalities (*p* = 0.005) and frontal balding (*p* = 0.015). Male patients more frequently had frontal balding (Risk ratio, 3.98; 95% confidence interval, 1.493–10.611) compared with female patients. Male patients with cataract presented more frequent cardiac abnormalities (Risk ratio, 4.40; 95% confidence interval, 1.055–18.358) compared with non-cataract male patients. Hypogonadism in male patients was characterized as decreased testosterone level, accompanied by elevated levels of luteinizing hormone and follicle-stimulating hormone.

**Conclusions:** In Chinese patients with DM1, we conclude that (1) cataract, hypogonadism, frontal balding and cardiac abnormalities are frequently observed; (2) age is an independent indicator to cataract and MIRS is the only predictor for cardiac abnormalities and frontal balding; (3) a positive correlation between ophthalmologic and cardiac impairments in male patients is found; (4) endocrine abnormalities show diverse manifestations and hormone tests are recommended; (5) particular attention should be given to patients with older age and higher MIRS score.

## Introduction

Myotonic dystrophy type 1 (DM1) is the most common muscular dystrophy in adults caused by an unstable CTG repeat expansion in the myotonic dystrophy protein kinase (*DMPK*) gene on chromosome 19q13.3 (1, 2). Individuals unaffected with DM1 have 5 to 37 CTG repeats. Individuals with 38 to 49 CTG repeats are asymptomatic but at risk of having children with larger expanded repeats. In DM1 patients, the number of CTG repeats increases to 50 up to thousands. The disorder is considered an RNA-dominant disease in which mutant transcripts containing an expanded CUG repeats are retained in nuclear foci and cause numerous dysfunctions by interfering with the regulation of alternative splicing (3). This multisystemic disease is characterized by myotonia, progressive muscle weakness, and atrophy, as well as the ocular, heart, endocrine system, respiratory function, and central nervous system-related abnormalities (4, 5). Patients could be asymptomatic or present mild late-onset adult to severe, but less prevalent congenital, form. The age of onset is inversely correlated with CTG repeat length (5).

Like in other neuromuscular disorders, muscle dysfunction in DM1 often results in significant disability (4, 6). Furthermore, multisystemic impairments such as cataract, cardiac abnormalities, hypogonadism, thyroid dysfunction, diabetes, or respiratory involvement have an additional impact on the quality of life, even causing a reduced life expectancy, mainly due to respiratory or cardiac failures [e.g., (7–16)].

Nowadays, there are no curative or disease-modifying therapies to DM1. Management mainly focuses on genetic counseling, prevention of cardiopulmonary complications, and symptomatic treatments (17). Nevertheless, there is currently an increasing consensus on the necessity of a multidisciplinary approach to managing the DM1 in order to ameliorate patients' quality of life. Therefore, a comprehensive assessment of multisystemic impairments in DM1 will significantly contribute to better management of the disease. While previous literature has respectively related lung disease, cardiac abnormalities, cataract, abnormal endocrine function or central nervous system dysfunction to muscular disability, sex, or CTG repeat expansion [e.g., (18–25)], only a limited number of studies [e.g., (22, 26, 27)] have focused on the intercorrelation among the various organs involved.

In addition, DM1 is not well studied in the Chinese population. One study has firstly reported different clinical characteristics of 37 Chinese DM1 patients comparing to those of Caucasian heritage (28). However, multisystemic impairments have never been described in a larger cohort of Chinese patients with DM1.

Therefore, we performed a retrospective study on the cohort of Chinese DM1 patients using investigations routinely performed in concomitance with the diagnosis. Aims of our study were to assess the prevalence and intercorrelation among multiple organ impairments in Chinese DM1 patients and their relationships with other clinical features (age at onset, disease duration, and muscular disability).

## Materials and Methods

### Patients

We retrospectively reviewed medical records of 122 consecutive clinically diagnosed DM1 patients in the outpatient unit of the Department of Neuromuscular Disorders at the Chinese PLA General Hospital from September 2010 to December 2018. Patients were recruited according to the following criteria: (1) diagnosis of DM1 confirmed by molecular genetic testing (29) either in the subject or in a first-degree relative; (2) recent assessment (<1 month) of blood biochemical, muscular, ophthalmologic, cardiac, and endocrine functions. The study was performed in accordance with the Declaration of Helsinki and approved by the Ethics Committee of the Chinese People's Liberation Army General Hospital. All patients or their guardians had given consent for their information to be used for clinical research purposes.

### Genetic Testing

Genomic DNA was extracted from patients′ peripheral leukocytes. Triplet-primed PCR (TP-PCR) was performed to determine the large abnormal *DMPK* alleles (29, 30). The primers were: P1 (5′ sequence fluorescently labeled by 5-FAM): 5′-AGA AAG AAA TGG TCT GTG ATC CC−3′, P2: 5′-GAA CGG GGC TCG AAG GGT CCT TGT AGC CG−3′, P3R: 5′-TAC GCA TCC CAG TTT GAG ACG−3′, and P4CTG: 5′-TAC GCA TCC GAG TTT GAG ACG TGC TGC TGC TGC TGC T−3′. PCR products were then analyzed using capillary electrophoresis on PRISM 3100 (Applied Biosystems) and GeneMapper 3.7 software. Based on TP-PCR, the exact number of expansions could not be obtained if the length of expanded CTG repeats was above 100 repeats.

### Assessments

The following data were recorded: sex; age at onset; age; disease duration; initial symptom; family history; muscular strength; score of Muscular Impairment Rating Scale (MIRS); serum levels of creatine kinase, glucose, testosterone, follicle-stimulating hormone (FSH), luteinizing hormone (LH), progesterone, free triiodothyronine (FT3), free thyroxine (FT4), and thyroid-stimulating hormone (TSH) respectively; results of surface 12-lead electrocardiogram, echocardiography, electromyography, and ophthalmologic examination.

Muscle strength was scored on a Medical Research Council (MRC, 0–5 point) scale using a manual muscle testing of 11 muscle groups bilaterally (31): the neck flexors, six proximal muscle groups (shoulder abductors, elbow flexors, elbow extensors, hip flexors, knee extensors, knee flexors), and four distal muscle groups (wrist extensors, digits flexors, ankle dorsiflexors, ankle plantar flexors). The MIRS (31), an ordinal five-point (1-5) rating scale, was performed by two neurologists (Zhanjun Wang and Mao Li) to assess the severity of muscular weakness: grade 1 = no clinical muscular impairment; grade 2 = early muscular impairment (clinical myotonia, facial weakness, and weakness of neck flexors) without limb weakness; grade 3 = distal weakness; grade 4 = mild to moderate (3 ≤ MRC score < 5) proximal weakness; grade 5 = severe (MRC score <3) proximal weakness.

Cataracts were visualized by slit-lamp examination and defined as lens opacities, from minimal to severe, disabling types.

Frontal balding was included as one aspect of skin impairment.

Cardiac abnormalities included: left ventricular ejection dysfunction (LVED) (if left ventricular ejection fraction <50% by echocardiogram); cardiac conduction disorders, including any atrioventricular or intraventricular blocks at surface 12-lead electrocardiogram; cardiac rhythm disorders, including premature atrial/ventricular contraction, atrial/ventricular fibrillation or atrial/ventricular tachycardia at surface 12-lead electrocardiogram.

Endocrine dysfunctions were defined as follows: (1) insulin insensitivity: impaired fasting glucose (IFG) or impaired glucose tolerance (IGT) if plasma glucose (PG) was between 6.1~6.9 mmol/L or 2 h PG between 7.8~11.0 mmol/L; diabetes if PG was ≥ 7.0 mmol/L or 2 h PG≥ 11.1 mmol/L; (2) hypogonadism if patients presented with erectile dysfunction (ED), testicular atrophy, azoospermia, habitual abortion, infertility or amenorrhea, with or without abnormal serum level of testosterone, FSH, LH, progesterone. To males, normal values from our hospital were 8.4–28.7 nmol/L for testosterone, 1.5–9.3 mIU/mL for LH, and 1.4–18.1 IU/L for FSH. Patients with decreased testosterone and/or increased LH/FSH were classified as absolute hypogonadism; those with normal testosterone and increased LH/FSH were classified as compensated hypogonadism. To females, because blood samples were collected at different stages in the reproductive cycle, normal upper levels of testosterone, LH, FSH, and progesterone were not able to be defined; (3) thyroid dysfunction if serum level of FT3, FT4 or TSH was abnormal.

### Statistical Analysis

Statistical analysis was performed using IBM SPSS software version 20.0 and included: (1) descriptive statistics concerning clinical data were applied to present as mean ± standard deviation (SD), median (M) with interquartile range (IQR), as well as minimum and maximum values or frequency with 95% confidence interval (CI); (2) for group comparisons: Mann–Whitney *U*-test, Student's *t*-test or Pearson's Chi-squared test were applied for numeric or categorical variables respectively; Risk ratio (RR) with 95%CI was used to express the difference between categorical variables; (3) association analysis between different variables was performed using Spearman's or Pearson correlation tests, as appropriate; (4) logistic regression analysis was applied to investigate the multivariate influence on multiple organ impairments; (5) RR was significant when 95%CI boundary values did not overlap value 1. The level of significance was set at *p* < 0.05.

## Results

Of the 122 medical records, 29 patients (18 males and 11 females) did not meet the criteria and were excluded. Ninety three DM1 patients from 88 families were included for analysis, comprising 60 males, aged 31.7 ± 12.3 years (mean ± SD; range, 0.5–59 years) and 33 females, aged 40.0 ± 11.3 years (mean ± SD; range, 18–67 years). The mean age of disease onset was 26.6 ± 12.4 years (mean ± SD). Median disease duration was six years (range, 0–28 years). Among the primary symptoms present at the onset, muscle weakness was observed in 47 cases (50.5%; 95%CI, 0.402–0.609), followed by myotonia (44.1%; 95%CI, 0.338–0.544) and slurred speech (2.2%). A majority of the patients were males. Male patients were younger and more frequently had frontal balding and hypogonadism compared with female patients. Clinical characteristics split by sex were summarized in Table 1.

**Table 1:** Summary of available clinical data on the DM1 cohort of study.

	***N*** **(%)**		**Mean (SD) or M (IQR)^a^**		***p*-value**	**RR of M/F (95%CI)**
	**M**	**F**	**M**	**F**		
Age (years)			31.7 (12.3)	40.0 (11.3)	<0.05	
Onset (years)			23.8 (11.3)	31.8 (13.0)	<0.05	
Duration (years)			6.5 (3.0-10.8)^*a*^	5.0 (2.0-14.0)^*a*^	NS	
Initial symptoms						
Weakness	26 (43.3)	21 (63.6)			NS	0.44 (0.182-1.047)
Myotonia	30 (50.0)	11 (33.3)			NS	2.00 (0.827-4.837)
Slurred speech	2 (3.3)	0 (0.0)				
MIRS			3.26 (0.72)	3.42 (0.83)	NS	
CK (U/L)			335.3 (147.8)	265.2 (233.3)	NS	
Increased level of CK	40 (83.3)	16 (55.2)			<0.05	4.06 (1.415-11.661)
Cataract	16 (37.2)	14 (56.0)			NS	0.47 (0.171-1.269)
Frontal balding	30 (51.7)	7 (21.2)			<0.05	3.98 (1.493-10.611)
Conduction disorders	14 (25.5)	6 (20.7)			NS	1.31 (0.443-3.870)
Rhythm disorders	5 (9.1)	4 (13.8)			NS	0.63 (0.154-2.534)
Insulin insensitivity	7 (14.3)	3 (11.5)			NS	1.28 (0.301-5.420)
Thyroid dysfunction	6 (14.0)	3 (14.3)			NS	0.97 (0.218-4.343)
Hypogonadism	25 (48.1)	6 (25.0)			0.057	2.78 (0.951-8.116)

a *M (IQR), NS, not significant; M, male; F, female*.

### Clinical Phenotypes

Ninety one patients had a juvenile or adult-onset of the disease, whereas only two cases, from two different pedigrees, were affected by the congenital DM1 (CDM).

**Figure 1:**
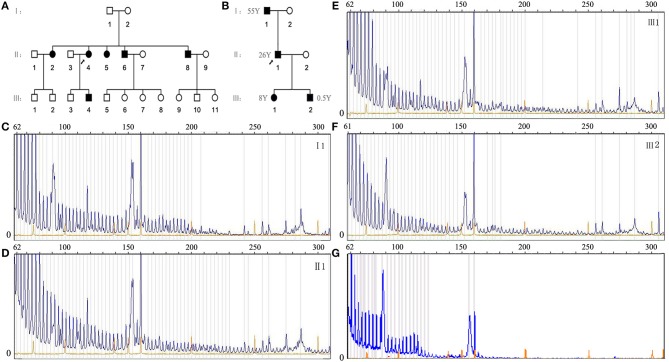
**(A)** Family chart of the maternally transmitted pedigree (black arrow indicating the proband); **(B)** family chart of the paternal transmitted pedigree (black arrow indicating the proband); **(C–G)** electropherogram results of TP-PCR for affected members (I1, II1, III1, III2) in the paternal transmitted pedigree and an unaffected individual with two alleles <30 repeats. X-axis illustrates the size in base pairs and Y-axis illustrates the allele peak height. The blue peaks, which start at the size of ~62, indicate expanded alleles. Yellow peaks are sizing ladders, included as inner reference. Blue peaks forI1 fade away at the size of ~216, showing the CTG repeat number is ~50. Blue peaks for II1, III1, and III2 all exceed the size of 305, showing the CTG repeat numbers are over 80. The large bands observed at ~ 90, 150, and 160 bp are also observed in the unaffected individuals with two alleles <30 repeats **(G)**, indicating bands at these positions are non-specific during PCR.

In the first pedigree (Figure 1A), patient III4 was a 7-year-old male who had a reduced fetal movement before birth. After delivery, he presented significant facial diplegia, which was characterized by an inverted V-shaped upper lip and cryptorchidism, which required surgical intervention. As he grew up, he also demonstrated intellectual disability with delayed development. Though no obvious myotonia and limb weakness were found, the EMG showed myotonic discharges accompanied by myopathic motor unit potentials. The patient's mother II3 (the 36-year-old proband), had experienced limb weakness and grip myotonia for 20 years.

In the second pedigree (Figure 1B), patient III2 was a 6-month-old male. His grandfather I1 was a 55-year-old asymptomatic patient containing ~50 CTG repeats (Figure 1C). Patient II1 was the 26-year-old proband, experienced grip myotonia and limb weakness for 17 years. Patient III1 was an 8-year-old female, experienced grip myotonia only for 6 months. Anticipation typically occurred in this paternally inherited pedigree (Figure 1B). Electropherogram results of TP-PCR for affected members (I1, II1, III1, III2) and an unaffected individual with two alleles <30 repeats were shown, respectively (Figures 1C–G).

### Muscular Impairment

**Figure 2:**
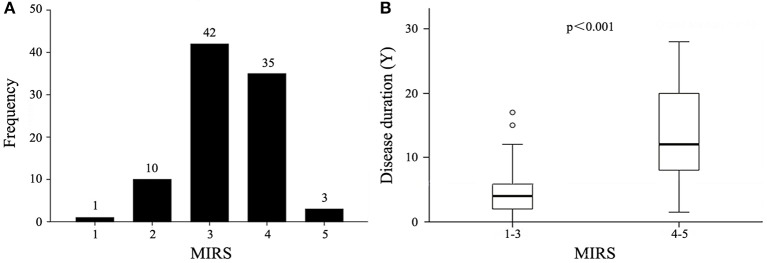
**(A)** Distribution of MIRS scores in 91 DM1 patients. **(B)** Longer disease duration with increasing MIRS. The horizontal line in the box represents the median. The bottom and top of the box represent the 25th and 75th percentile, respectively. The error bars represent the range.

Apart from the two Congenital patients, 91 patients were categorized into the 5 MIRS classes (Figure 2A). The mean MIRS score was 3.3 ± 0.8 (mean ± SD). MIRS was positively correlated with age (*r* = 0.44, *p* < 0.001) and disease duration (*r* = 0.64, *p* < 0.001), but not significantly correlated with onset age (*p* = 0.561). Upon multivariate analysis with inclusion of age, sex, and disease duration as co-factors, MIRS was mainly correlated with disease duration (*p* < 0.001) and more weakly correlated with age (*p* = 0.016). Patients categorized as MIRS 4, 5 had a longer duration than those categorized as MIRS 1, 2, 3 (*p* < 0.001) (Figure 2B). A comparison of other clinical parameters divided by severity of the muscular disability was shown in Table 2. 72.7% (56/77; 95%CI, 0.626–0.829) of the patients with DM1 had increased levels of creatine kinase (CK). The level of CK was not positively correlated with MIRS (*p* = 0.531).

**Table 2:** Summary of clinical data in DM1 patients divided by severity of muscular disability.

	**MIRS 1-3**		**MIRS 4-5**		***p*-value**	**RR of MIRS 4-5/1-3 (95%CI)**
	**%**	**Mean (SD)**	**%**	**Mean (SD)**		
Age (years)		31.8 (11.4)		40.2 (10.7)	<0.05	
Onset age (years)		27.2 (11.8)		27.2 (12.2)	NS	
Disease duration (years)		4.0 (2.0-6.0)^a^		12.0 (8.0-20.0)^a^	<0.05	
Sex (male)	67.9		57.9		NS	0.65 (0.274-1.541)
Creatine kinase (U/L)		288.9 (135.8)		331.7 (231.0)	NS	
Increased level of CK	75.6		69.4		NS	0.73 (0.268-2.004)
Cataract	35.7		57.7		NS	2.46 (0.901-6.684)
Frontal balding	32.1		52.6		0.49	2.35 (0.997-5.556)
Cardiac abnormalities	25.5		48.5		<0.05	2.75 (1.087-6.964)
Conduction disorders	15.7		36.4		<0.05	3.07 (1.090-8.652)
Rhythm disorders	9.8		12.1		NS	1.27 (0.315-5.118)
Insulin insensitivity	12.8		14.3		NS	1.14 (0.292-4.445)
Thyroid dysfunction	7.5		25		NS	4.11 (0.921-18.351)
Hypogonadism	33.3		53.6		NS	2.31 (0.888-5.996)

a *M (IQR), NS, not significant*.

### Extramuscular Disorders

**Figure 3:**
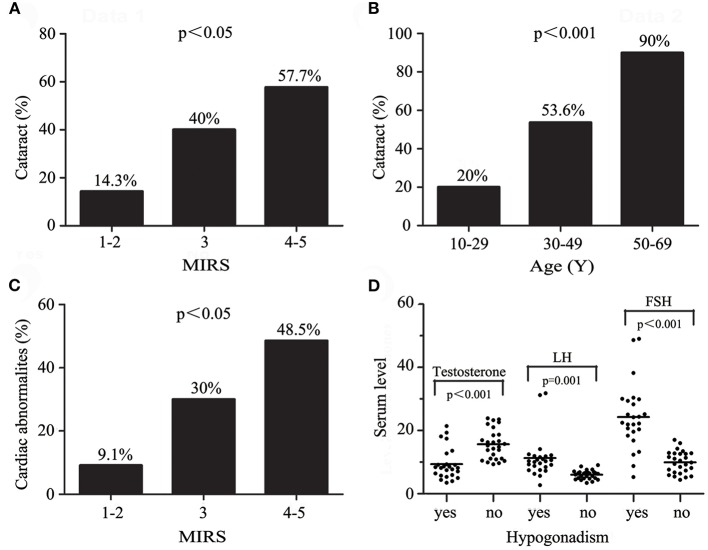
**(A)** The prevalence of cataract increases with MIRS. **(B)** The prevalence of cataract increases with age. **(C)** Progression of cardiac abnormalities with increasing MIRS. **(D)** Comparison of testosterone, LH and FSH levels in male patients with or without hypogonadism.

44.1% (30/68; 95%CI, 0.320–0.562) of patients, aged 42.9 ± 11.9 (mean ± SD) years, presented with cataract, characterized as bilateral iridescent or whitish, multiple, dust-like opacities in the posterior subcapsular layer of the lens (17/30; 95%CI, 0.378–0.755), cortex of the lens (5/30; 95%CI, 0.025–0.308) or lens with undetermined distribution (8/30; 95%CI, 0.099–0.435). Most patients did not express complaints of obvious vision loss, except two individuals who underwent surgery for a disabling cataract. The presence of cataract was correlated with MIRS (*r* = 0.27, *p* = 0.028) (Figure 3A) and age (*r* = 0.55, *p* < 0.001) (Figure 3B), but not correlated with disease duration (*p* = 0.075). Patients with cataract were characterized by more advantaged age (mean ± SD: 42.9 ± 11.9 years vs. 29.1 ± 8.8 years; *p* < 0.001). Logistic regression analysis with age, sex, and MIRS as co-variables only found age (*p* < 0.001, Hosmer and Lemeshow test 0.197) as an independent factor in explaining cataract.

40.7% (37/91; 95%CI, 0.304–0.509) of patients, aged 35.0 ± 10.2 (mean ± SD) years, presented with signs of frontal balding, 30 of which were male. Male patients and patients with MIRS 4–5 had a risk of frontal balding 3.98 times higher (95%CI, 1.493–10.611) and 2.35 times higher (95%CI, 0.997–5.556), respectively than the other patients. Logistic regression analysis showed sex (*p* = 0.002) and MIRS (*p* = 0.015) as independent factors in explaining frontal balding (Hosmer and Lemeshow test 0.882). The level of testosterone in male patients with or without frontal balding was 12.2 ± 6.2 nmol/L vs. 13.1 ± 5.3 nmol/L (mean ± SD, *p* = 0.577).

Echocardiogram findings of LVED were observed in none of 51 patients. Cardiac conduction disorders and rhythm disorders were found in 23.8% (20/84; 95%CI, 0.145–0.331) and 10.7% (9/84; 95%CI, 0.040–0.175) of patients, respectively (Table 3). Cardiac abnormalities, found in 34.5% (29/84; 95%CI, 0.241–0.449) of patients, were more frequent in patients with older age (mean ± SD: 37.9 ± 12.3 years vs. 32.5 ± 10.7 years; *p* = 0.039) and higher MIRS (mean ± SD: 3.6 ± 0.7 vs. 3.1 ± 0.7; *p* < 0.01) (Figure 3C) than the others. With age, sex, and MIRS as co-variables, a correlation was only found between cardiac abnormalities and MIRS (*p* = 0.005, Hosmer and Lemeshow test 0.518) (Table 2).

**Table 3:** Types of cardiac abnormalities in patients with DM1.

	***N* (%)**
Cardiac conduction disorders	20 (23.8)
First-degree atrioventricular block	9 (10.7)
Right bundle branch block	4 (4.8)
Left anterior fascicular block	1 (1.2)
Other intraventricular blocks	6 (7.1)
Cardiac rhythm disorders	9 (10.7)
Tachycardia	3 (3.6)
Premature contraction	6 (7.1)

14.1% (9/64; 95%CI, 0.053–0.228) of patients showed thyroid dysfunction. Six patients had hypothyroidism or subclinical hypothyroidism characterized as elevated TSH level with or without a decreased FT4 level. Hyperthyroidism was found in the other three patients. MIRS degree, age, sex, or disease duration did not differ between patients with or without thyroid dysfunction. Insulin insensitivity was found in 13.3% (10/75; 95%CI, 0.055–0.212) of patients, who were older (mean ± SD: 42.1 ± 5.0 years vs. 33.5 ± 12.9 years; *p* = 0.001) than patients without insulin insensitivity. We did not find any relationship between MIRS, sex or disease duration, and insulin insensitivity.

Hypogonadism was found in 48.1% (25/52; 95%CI, 0.340–0.621) of male patients. 9.6% (5/52; 95%CI, 0.013–0.179) of those had erectile dysfunction (ED), 21.2% (11/52; 95%CI, 0.097–0.326) had compensated hypogonadism and another 26.9% (14/52; 95%CI, 0.145–0.394) had absolute hypogonadism. Males with hypogonadism tended to be older (mean ± SD: 35.2 ± 10.9 years vs. 29.4 ± 10.4 years; *p* = 0.054), having a lower level of testosterone (mean ± SD: 9.3 ± 5.0 nmol/L vs. 15.6 ± 4.7 nmol/L; *p* < 0.001) as well as higher values of LH (mean ± SD: 11.2 ± 6.6 mIU/mL vs. 5.9 ± 1.4 mIU/mL; *p* = 0.001), FSH (mean ± SD: 24.2 ± 10.4 IU/L vs. 9.8 ± 3.5 IU/L; *p* < 0.001) and FSH/LH ratio (mean ± SD: 2.3 ± 0.6 vs. 1.7 ± 0.7; *p* < 0.01) (Figure 3D) than males without hypogonadism. There was also a close positive correlation between LH and FSH levels (*r* = 0.83, *p* < 0.001) in male patients. The mean LH, FSH and testosterone levels divided by severity of muscular disability were shown in Table 4. No correlation was found between hypogonadism and MIRS, or disease duration in male patients. 25% (6/24; 95%CI, 0.063–0.437) of female patients presented with infertility or irregular menstruation, accompanied by decreased progesterone.

**Table 4:** LH, FSH, and testosterone levels in male patients divided by severity of muscular disability.

	**MIRS 1-2**	**MIRS 3**	**MIRS 4-5**	***p*-value**
	**Mean (SD)**	**Mean (SD)**	**Mean (SD)**	
LH (mIU/mL)	6.6 (3.2)	7.6 (2.4)	10.5 (8.1)	NS
FSH (IU/L)	13.7 (9.2)	15.8 (8.4)	19.4 (13.4)	NS
Testosterone (nmol/L)	12.8 (6.7)	13.2 (5.9)	11.6 (5.3)	NS

*NS, not significant*.

### Intercorrelations Among Different Organs Involved

Patients with cataract tended to more frequently have cardiac abnormalities (42.3 vs. 21.1%, *p* = 0.068) and hypogonadism (41.7 vs. 24.2%, *p* = 0.162) compared with non-cataract patients, although the results were not statistically significant. When stratified by sex, male patients with cataract presented more frequent cardiac abnormalities (RR, 4.40; 95%CI, 1.055–18.358) and hypogonadism (RR, 3.83; 95%CI, 0.963–15.187) compared with non-cataract male patients. Logistic regression analysis revealed that age (*p* = 0.002, Hosmer and Lemeshow test 0.957) explained these relationships. No statistical relationships could be observed between any of the other two extramuscular organs involved.

## Discussion and Conclusions

In China, DM1 has not been well studied for a long time and is considered a less prevalent disease in the population compared to that of Caucasian and Japanese (32), which could be due to a low founder effect or lack of asymptomatic or atypical cases ascertainment (33). Until now, there is a considerable gap in studies about DM1 in the Chinese population. Hence, we report the frequency of extramuscular disorders and analyze the intercorrelation among multiple organ impairments in the largest cohort of Chinese patients with DM1 so far reported in the literature.

Of the 93 DM1 patients, two cases are congenital forms. One patient from the first pedigree is maternally transmitted. He has survived his prenatal and neonatal periods, with the typical “tented” upper lip as well as delayed development. His muscle strength remains intact at that time, which fits the feature of CDM (34). Though cryptorchidism is rarely reported as a complication of CDM, Zapata et al. (35) reported a frequency of 42% of occurrence in male CDM. The other congenital patient from the second pedigree is paternally transmitted. In the literature, paternally inherited CDM is unusual, and near all fathers of CDM have small repeat sizes and/or were asymptomatic at the time of their child's birth (36). In our study, the paternally inherited CDM's father is a classical patient with DM1, though his grandfather is asymptomatic, containing ~50 CTG repeats. The complication of cryptorchidism and anticipation with paternal transmission both show us characteristics less commonly seen in CDMs before.

In the present study, patients with MIRS grade 3 were most common, and 87.9% (80/91) of all DM1 patients had MIRS≥3, indicating that some early symptoms like myotonia or facial weakness might go disregarded, and only disabling symptoms like distal or proximal weakness could ultimately lead to hospitalization. Our results show the MIRS mainly correlates with increasing disease duration, consistent with the fact that MIRS reflects the natural history of DM1 progression (31). Based on present data, it possibly requires, on average, eight years for a DM1 patient with mild muscular symptoms to deteriorate into moderate and severe muscular disability. Relationships between multiorgan disorders and MIRS are discussed below.

A cataract is the most common extramuscular disorder in DM1 (37). On slit-lamp examination, it appears typically as bilateral posterior subcapsular or cortical iridescent lens opacities. The co-existence of cataract and myotonia is often suggestive of DM1. In our study, the majority of patients with cataract do not have complaints of vision impairment, making it necessary for the use of slit-lamp examination. The multivariate analysis revealed that the presence of cataract in DM1 increases specifically with age, not with disease duration or MIRS, indicating that the aging itself might serve as a predicting factor for the presence of cataract. In patients aged between 50 and 69 years old, the prevalence of cataract is up to 90% (9/10). As for the relationship between cataract and MIRS, the correlation is not strict (37), despite Kaminsky et al. have reported MIRS and age as independent factors in correlating with cataract.

Cardiac abnormalities are a major prognostic factor in patients with DM1. Surface 12-lead electrocardiogram is recommended for the prediction of adverse cardiac events (38) in DM1 patients, including those without cardiac symptoms. Cardiac abnormalities of DM1 can be divided mainly into conduction disorders, rhythm disorders and cardiomyopathy. In our study, a first-degree atrioventricular block is the most common electrocardiogram abnormality, which is inconsistent with most studies, but the prevalence is lower (10.7 vs. 40%, 23.6%) (39, 40). The decreased prevalence happens when it comes to rhythm disorders and LVED (10.7 vs. 20.8%, 15.5 and 0% vs. 12.3%) (22, 40). Otherwise, atrial fibrillation or other malignant arrhythmia is not found in our patients, which could attribute to the un-use of 24 h Holter electrocardiogram monitoring. Previous reports have shown age, male, and muscular disability have a correlation with cardiac abnormalities (22, 41, 42). Partially consistent with these reports, we found that increasing age and MIRS correlate with a higher prevalence of cardiac abnormalities in DM1 patients. However, only MIRS is the independent predictive factor. In patients with MIRS 4-5, the prevalence of cardiac abnormalities is up to 48.5% (16/33). In contrast, the rate is only 9.1% (1/11) in patients with MIRS 1-2.

Recent findings show that insulin resistance and hypogonadism are the main endocrine dysfunctions in DM1 (37, 43). In contrast, thyroid dysfunction is less reported. In the present study, thyroid dysfunction has a similar low frequency with insulin resistance (14.1 vs. 13.3%). Hypothyroidism and hyperthyroidism both found in one group of DM1 patients has been reported before (23), which suggests a complex mechanism of thyroid dysfunction in DM1.

Hypogonadism is the second most frequent extramuscular disorder in our study. Erectile dysfunction is only found in 9.6% of male patients, and the frequency is much lower than that of 72% in a Serbian report (χ^2^ = 31.367, *p* < 0.001) (44). In agreement with previous studies (23, 44, 45), hypogonadism in male patients tends to have a typical pattern of gonadal hormones, characterized as decreased testosterone as well as elevated FSH, LH, and FSH/LH ratio. But we fail to demonstrate a significant correlation between hypogonadism and severity of muscular disability (45), though levels of FSH and LH were higher in patients with MIRS 4–5 than in patients with MIRS 1–2. To females, hypogonadism is much less frequent and maybe underdiagnosed because the level of gonadal hormones is difficult to assess as it fluctuates with the menstrual cycle (23). For this reason, data of hypogonadism in females are not used for most statistical analyses.

Alopecia, usually called frontal balding, has been reported as a prominent feature of DM1, with a prevalence between 33.3 and 55% (46–48). In accordance with the previous study, we found that 40.7% of patients had frontal balding, whose prevalence is significantly higher, but not exclusive in male patients. Alopecia in DM1 is often known as an androgen-dependent disorder, whose serum androgen levels tend to be lower than normal, indicating the peripheral response to androgens is more important than absolute circulating levels (49). Our data, which found no difference at the hypogonadism level in male patients with or without frontal balding, to some extent, may support this proposition. Observational studies are still needed to assess other skin impairments, such as pigmented lesions (nevi), epithelial tumors (pilomatrixoma, dermatofibroma), and inflammatory dermatoses (xerosis, seborrheic dermatitis), which are reportedly common in DM1 patients.

We did not find correlations between any of the two organs involved, except the correlation between cataract and more frequent cardiac abnormalities (RR, 4.40; 95%CI, 1.055–18.358) compared with non-cataract in male patients. This finding indicates that sex could be considered as a factor in the assessment. Dogan et al. reported the impact of sex on DM1 in French by showing the unequal prevalence of several DM1 symptoms between men and women (25). In the present study, the sample included an excess of younger males in prevalence with a higher frequency of frontal balding and hypogonadism. In order to eliminate a potential bias caused by sex and age, a multivariable analysis was used to show only the frontal balding is more frequent in male patients.

The main limitations of this study are related to its retrospective nature, its lack of ability to provide CTG repeat lengths for analysis, and lack of data referring to pulmonary or other common extramuscular dysfunctions. In our work, assessment of respiratory impairment was based predominantly on whether patients presented with dyspnea or orthopnea, which is not enough to illustrate respiratory involvement in DM1. Only two patients, both categorized as MIRS 5, presented with exertion dyspnea and orthopnea. One of them had already started Non-Invasive Ventilation (NIV) following the instructions from pulmonologists. Next, we will include other symptoms such as insomnia, morning headaches, snoring, excessive daytime sleepiness, and history of chest infection in the symptom checklist and to use pulmonary function tests as a better assessment of respiratory impairment in DM1. In addition, the most commonly used genetic analysis tool in China, a semi-quantitative PCR (TP-PCR), is unable to determine an exact number of expansions if the length of expanded CTG repeats is above 100 repeats. Due to this limitation, we were unable to use CTG expansion size as a predictor of the severity of clinical manifestation in DM1, but to find an alternative indicator based on clinical data.

In conclusion: we demonstrate a frequent prevalence of cataract, hypogonadism, frontal balding, and cardiac abnormalities in Chinese DM1 patients. We also show a positive correlation between ophthalmologic and cardiac impairments in male patients, whereas particular attention should be given to older DM1 patients with higher MIRS scores.

## Data Availability Statement

The datasets generated for this study are available on request to the corresponding author.

## Ethics Statement

The studies involving human participants were reviewed and approved by Ethics Committee of the Chinese People's Liberation Army General Hospital. Written informed consent to participate in this study was provided by the participants' legal guardian/next of kin.

## Author Contributions

ML: data interpretation and drafting of the manuscript. ML, ZW, and FC: data collection and data analysis. FY and HW: genetic testing. XH: critical revision and final approval of the manuscript. All authors agree to be accountable for the content of the work.

## Conflict of Interest

The authors declare that the research was conducted in the absence of any commercial or financial relationships that could be construed as a potential conflict of interest.
